# Artificial Procidentia Uteri, as a Means of More Effectual Treatment of Cancer of That Organ

**Published:** 1868-02

**Authors:** E. F. Gordon

**Affiliations:** Mobile, Ala.


### ARTIFICIAL PROCIDENTIA UTERI, AS A MEANS OF MORE EFFECTUAL TREATMENT OF CANCER OF THAT ORGAN.

By E. F. GORDON, M.D., Mobile, Ala.

In proposing such an extreme plan, I am aware that I am running counter to the experience of distinguished men, and to the present teachings of surgery and medicine.

Let us for a moment review the history of the course and treatment of cancer of the womb, as we ordinarily meet with it. We are summoned to take charge of these cases usually, when the pains, hemorrhages, and putrid discharges alarm our patients.

It is the fate of our laboring classes that, as their hardier frames bear pain better, we rarely see them until the disease is far advanced. The two forms of carcinoma, fibrous and encephaloid, are unfortunately common, and the latter strides on with fearful rapidity. The epithelial, first described by Sir Charles Clark, as the cauliflower excrescence, pours out at an earlier period a profuse serous discharge, oftentimes acrid, but not at that stage offensive, and in this way, and from the fact of its slower march, is oftener seen by the physician while yet confined to the original cervix.

It is unnecessary here to enter into the pathology of this neoplasm. Whether it is, according to Chambers' view, a defect of vital force which equally predisposes to the deposition of tuberculous and cancerous matter, by degeneration of the blood, it is only too well known that its progress is always towards destruction.

Those of us who are in daily contact with this disease, are familiar with its loathsome train of symptoms, and with the inevitable result, sooner or later, but generally after two years of terrible suffering.

The standard authorities advise for treatment that we endeavor to improve the general health by bitter tonics, and to confine ourselves to allaying pain by anodynes, checking hemorrhages by astringents and the tampon, and keeping down offensive odors by chlorinated washes. In former times, starvation was occasionally practised, and an exclusive vegetable diet was recommended by Dr. Twitchell of New England, from the result of a case under his treatment, but I fear it only adds to the broken-down condition of the patient.

Internally, all the more powerful articles of the Materia Medica (arsenic hardly yet abandoned) have been tried and found wanting. Locally, the actual cautery, Vienna paste, chloride of zinc, chromic acid, bromine, and latterly the solvents of cancer cells, such as acetic, citric, and carbolic acids, have been, by turns, the favorite, but have failed in the end to justify the anticipations of their advocates. Excision of the cervix has been tried extensively by the French surgeons, and in a more restricted way by others. But the task is so hopeless, and so dark with painful forebodings, that we shudder when we are summoned to take charge of such a case, and to bid our patients, like the Third Richard, "despair and die."

It is with a desire of abridging such scenes as this, and, if possible, to place the uterus in a position where it can be daily inspected and rationally treated, that I have to propose the induction of procidentia by artificial means.

Is there anything in the anatomical relations of the uterus to forbid the effort? Bear with me while I condense a statement of these relations. This organ is attached to the bladder and rectum by folds of the peritoneum, which are sometimes called the anterior and posterior ligaments. The peritoneum reflected upwards covers the anterior and posterior surfaces of the uterus enclosing the organ between two of its layers. These layers meet together at the sides of the uterus, and pass off to the lateral walls of the pelvic cavity, dividing the pelvis transversely.

In this way the peritoneum forms the principal part of the broad ligaments. The fibrous or muscular structure of the uterus itself also extends into these ligaments. They contain besides the Fallopian tubes, the ovaria, the round ligaments, with bloodvessels, nerves, and lymphatics. The round ligaments arise from the upper angles of the uterus in front of the Fallopian tubes. From this origin, each ligament passes to the inguinal ring, descends the inguinal canal, turning round the epigastric artery, and its fibres are inserted into, or are mixed with, the structures of the mons veneris. The length of the round ligaments is from four to five inches.

The external traverse fibres of the proper substance of the uterus are prolonged into the round ligaments, of which they form a constituent part. Some fibres of the internal oblique muscle also enter the lower part of the canal, and extending upwards, contribute to the formation of the ligament. The ovaria are connected with the uterus at the point of insertion of the Fallopian tubes by a fibro-cellular cord or ligament, prolonged from the proper substance of the uterus (Tyler Smith). The womb rests upon the upper end of the vagina, which encloses its cervical or neck portion, and keeps it up in its place, by means of its connection with the bladder in front and the rectum behind, and more than all, by two utero-sacral ligaments, which tie the upper ends of the vagina and womb to a certain place about an inch and a-half in front of the apex of the sacrum. As long as the utero-sacral ligaments remain in a healthy state, preserving by their tone a due length, the womb cannot fall downwards or prolapse, because the cervix, being enclosed within the upper end of the canal of the vagina, cannot move down unless the upper end of the vagina move down also. Prolapse pulls upon and stretches all the ligaments—the broad ligaments by far the most. The vagina suffers displacement proportionally with the prolapse. It is inverted, its walls being doubled upon themselves, and its cavity progressively shortened until it is entirely effaced.

This displacement is always in a direction corresponding with the axis of the vagina and different portions of the pelvis, and follows the curve formed by the hollow of the sacrum and continued by the perineum (Byford). There is nothing then to prevent prolapsus of the uterus but the tonicity of the ligaments suspending it, and holding it from above, and the vagina supporting it from below. When the uterus is enlarged and presses down, and when the ligaments are relaxed and weakened by long-continued strain, or an impoverished condition of the system, prolapse is apt to occur *spontaneously*.

"Does the disease itself (cancer) change the parts so early as to make the effort dangerous?"

There is a singular unanimity among writers as to the part in which uterine cancer begins, and from which it extends itself. Prof. Rokitanski, of Vienna, whom Simpson pronounces "perhaps the most experienced and profound morbid anatomist of the present day," says, "carcinomatous induration generally limits itself to the vaginal portion and cervix, and very often in a defined and sharp manner." In another paragraph, he remarks, "the primitive seat of cancer is always the cervix uteri, and first of all and particularly the vaginal portion. The primary appearance of cancer in the fundus uteri is limited to so extremely rare cases, that what we have just said remains one of the *most fixed rules*. It forms," he adds, "in this respect, a contrast with fibrous and tuberculous tumors of the uterus."

"Uterine cancer," says Prof. Walshe, a very high authority, "is commonly primary, and possessed of comparatively slight tendency to contaminate the system generally." And again, "there can be no question that the womb ranks among those organs less prone than certain others, as, for instance, the mammæ and testes, to contaminate the distant viscera. Among thirty-seven females dying of uterine cancer, and examined by M. Ferrus, *seven* only exhibited secondary formations elsewhere." Scanzoni remarks, and his experience is worthy of attention, for he has tabulated one hundred and eight cases treated by himself in eight years: "Among all the parts of the uterus, it is almost exclusively the vaginal portion which is the starting point of the disease; in fact, although observations exist of cancers which are developed in the body, or near the summit of the uterus, while the neck was entirely untouched, or elsewhere, the infiltration was equally spread throughout the organ, such cases ought to be considered as rare exceptions."

The progress of the growth is very generally from the neck upwards, and from the interior of the cavity outwards. It struggles on in this way for perhaps six months on an average, before the infiltrations extend to the vagina and peritoneum, and finally to the rectum, bladder, and inguinal glands. In younger persons, menstruation seems to hasten the development, but after its cessation, at the change of life, the advance is slower. It is plain then that we must limit our efforts to produce artificial procidentia to the early period of this disease, while yet the uterus is moveable, and before the peritoneum is involved, as we would risk producing intense peritonitis by tearing that membrane if diseased, besides the utter hopelessness of ultimate cure when the abdominal structures are already implicated.

At this stage, too, you avoid the hemorrhage incident to bruising the fungosities, which, at a later period, shoot from the neck. The cancroid or epithelial form presents the most hopeful aspect, from its later tendency to deep ulcerations and adhesions. Its character is well given by Virchow, who first recognized it in the list of papillary tumors. Mayes says it is not a cancer, but a peculiar excrescence of the female genital organs. At first, it is purely local and nowise constitutional, but after a time it assumes a cancerous character. The enlarged papillæ are covered with looped capillaries, which abundantly supply them with blood, and present that red strawberry appearance which bleeds on the slightest touch. Subsequently, cancroid deposits take place deeper among the muscular fibres and connective tissue. The scirrhus or fibrous comes next, and may, in a few instances, be seen early enough, but the encephaloid will be apt to have advanced so far as to baffle all our efforts, and to forbid the attempt to bring the organ down.

I proceed to the next point—the best plan of effecting procidentia. Two modes suggest themselves: one is to attach a wire to the cervix, by passing it around or through the latter, and by suspending a moderate weight to it, by which we may tire out the ligaments. This is open to the objection of being very slow and disagreeable, and confining the patient to the recumbent or sitting posture; and also that the wire might irritate or cut out if much traction was necessary. A gum-elastic cup and cord might be substituted, by which, the air being exhausted, a firm but slight pull could be kept up, without the inconvenience of the wire and weight.

The plan that seems to me most feasible, is to place the patient under the influence of chloroform, so as to obtain the double benefit of complete relaxation of the tissues, and the protection of the patient from the suffering and shock induced by the anæsthesia. By means of Museaux hooks, the uterus can be pulled down gently, following the axis of the pelvis and vagina until it reaches the vulva. This can be repeated two or three times a week, watching the effect on the patient, and endeavoring at each trial to protrude it a little further. In two months we might hope to establish complete procidentia, and accustom the patient to it, so as to avoid the sinking and nausea attendant on the operation. Large doses of opium such, as are used after ovariotomy and in puerperal peritonitis, might be resorted to for the purpose of establishing toleration, after the prolapse was obtained.

Is extirpation of the cervix uteri necessarily dangerous?

It will be remembered that this operation was first suggested in modern times, and performed by Osiander, of Gottingen, about the year 1822, and that soon afterwards it was brought into vogue by Lesfranc, who reported ninety-nine cases, with a loss, I think, of only fourteen.

This was in a formal dissertation submitted to the French Academy.

It will also be borne in mind that M. Panly, who was an interne at La Pitié at the time, proves conclusively that these statistics are entirely unreliable, many of the cases having been reported twice under different names, and a large number dying between the first and fourth day. Again, he furnishes a list of nineteen cases in private practice, where, under the same operator, the mortality was nearly one-half.

This would seem to make against rather than in favor of the operation; but when we consider that, at that day, the diagnosis of cancer was very imperfect, and that the cases were operated on without any reference to the stage of the disease, it is only a matter of surprise that so many of them should have recovered.

It would seem that the operation had never attained any popularity in Great Britain until revived by Simpson, who reports eight cases, six of them successful, and only one proving fatal. Three of these cases cured were cancerous.

So far as regards the dangers of the operation, Simpson remarks, that he has not met with troublesome hemorrhage, although using only the bistoury and scissors, and that the shock which is described as being so fearful, by many writers, did not occur in any of his cases.

Chaissaignac, since the invention of his ecraseur, has again popularized the operation in France, and must have performed it many hundred times, extending its application to hypertrophies of the neck and even ordinary prolapsus.

By his instrument all danger of hemorrhage is removed, and by the use of chloroform the shock is much lessened or entirely obviated.

Dr. J. Braxton Hicks says, "he has operated or been present in more than twenty-eight cases, and has never seen any fatal result or any untoward symptom whatever."—*Guy's Hospital Reports.*

Any one who glances over the medical journals of late years, is struck with the frequency of this operation and how little danger is attached to it, and all the systematic treatises now recognize it as justifiable, not excepting Scanzoni, who is extremely conservative and cautious in recommending surgical interference.

In point of fact, Sims' favorite operation of bifurcating the cervix for the cure of dysmenorrhœa is quite as severe, the incisions being as extensive, though in an opposite direction. I may add, that he seems to fear hemorrhage more than most surgeons, tamponining with perchloride of iron to prevent it, and cautioning against its removal or any exercise for three or four days.

Can the uterus be removed without a fatal result?

I do not wish to dispute the assertion that it is one of the most dangerous operations in surgery to attempt its removal *in situ.*

Statistics conclusively prove that out of nineteen cases quoted by Breslau, two only had succeeded—Langenbeck's and Recamier's—Sauteis is mentioned by some authors as a third, although the patient died in a few weeks after of a colic. Blundell performed the operation three times, only once succeeding. His method of operating, by retroverting the organ and pulling it out by Douglas' cul-de-sac, seems to me the most feasible. I saw an uterus after it was extirpated by Dr. Paul Eve, then of Augusta, Ga., in 1850. The patient lived for several months afterwards, but died subsequently from a return of the cancer.

Dr. Storer, of Boston, removed, last year, a large tumor, containing the womb and appendages, from a patient who is represented to have made a good recovery. In such a case you have many of the same difficulties to be overcome and dangers to encounter as in ovariotomy.

Apart from the cases in which nature has done more than half of the duties of the operation, by precipitating the organ out of the vulva; by isolating it from the important parts with which it is in relation in the ordinary state; by having also prepared the way, by long habitude, for the void which the absence of the uterus occasions in the pelvic cavity. Apart from these cases, in which the ablation is easy and little painful, *and attended with certain success*, the extirpation of the uterus constitutes one of the most frightful operations, even to the rashest surgeon, and is the most dangerous to the patient. This is the admission of Duparcque, while denouncing the operation, and is well worthy of our attention. For if spontaneous procidentia deprives this most terrible ordeal of all danger, then we ought to imitate Nature's plan of effecting such a displacement preparatory to the removal of the womb. Women have been known to cut off the prolapsed womb with an ordinary knife, and many surgeons have amputated the organ, to relieve patients from the inconvenience of the dragging and discharges, especially after inversion. Sims reports such a case in his late book, and Dr. Choppin, of New Orleans, relates a case of prolapsus in the February Number of the *Southern Journal of Medical Sciences*, cured by ablation. He jestingly remarks that the woman was afterwards presented to the Class "with her womb in her hand," thus demonstrating that the uterus could be removed without causing death.

The advantages, then, of treating uterine cancer by means of artificial procidentia are, that you can make your applications with more accuracy and certainty; that the vagina will be less excoriated by the discharges, and that adhesions cannot be formed in such a way as usually bind the womb above; that, day by day, you can watch the progress of the disease, and if you find it encroaching on the cervix, it can be cut off in a few moments by the scissors, or more slowly by the ecraseur; and, lastly, if the cancer still advances and implicates the body, a way has been prepared for its entire removal.

For I think the evidence is conclusive that toleration of the procidentia is equivalent to toleration of the operation for removal.

Another point here comes up, the justification of any or all surgical operations in cancerous tumors.

In 1850, I heard an able report read by Professor Mussey, of Cincinnati, against the propriety of such operations, founded on the experience of American surgeons, but I think quite a change has occurred since then in medical opinion. Mr. Birkett, of Guy's Hospital, London, published a paper in 1866, giving his experience in one hundred and fifty cases, covering a period of eighteen years. It is carefully prepared, and his deductions are given with great circumspection and are entitled to much respect. He sums up by saying: "In conclusion, I

trust that I have demonstrated to my skeptical professional brethren, that a certain proportion of cancer patients can receive benefit by submitting to the removal of the first growth of the disease, and that the benefit derived from the operation is two-fold, *viz.:* 1st, prolongation of life; 2d, exemption from disease for a considerable period of time in many instances."

It may seem irrelevant or superfluous to have discussed the surgical questions alluded to in this essay, but I do not think artificial procidentia justifiable, unless surgical interference in all its various grades may be resorted to with propriety.

To recapitulate. I have endeavored to show, 1st, that there is nothing in the anatomy of the parts to prevent the induction of procidentia, but that in fact it occasionally occurs spontaneously. 2d. That in the early stage of cancer, adhesions do not exist to such an extent as to render it dangerous or impracticable: and, 3d. That applications of caustic can be made more accurately to the prolapsed organ; and that amputation of the cervix, and, in extreme cases, ablation of the entire uterus, can be practised with safety under these circumstances.

I am aware that it may be said that all these operations can be performed without all this long preparation and infliction of suffering, but my own experience warrants me in saying that the old plan has invariably failed in our hands in arresting the disease, and anything that offers more chances of success should not be discarded without a fair trial.

I am sorry, however, to be obliged to confess that this paper was prepared about eight months ago, and, during the interval that has elapsed, I have not found a case in a sufficiently early state of development to enable me to apply the proposed method. —*New Orleans Journal of Medicine.*

